# No Evidence of Genetic Basis to Variation in Human Offspring Sex Ratio

**DOI:** 10.1007/s10519-026-10266-0

**Published:** 2026-05-05

**Authors:** Kaitlyn T. Harper, Ralf Kuja-Halkola, Karin J. H. Verweij, Patrik K. E. Magnusson, Brendan P. Zietsch

**Affiliations:** 1 Centre for Psychology and Evolution, School of Psychology, University of Queensland, Brisbane, Australia; 2 Department of Medical Epidemiology and Biostatistics, Karolinska Institutet, Stockholm, Sweden; 3 Department of Psychiatry, Amsterdam UMC, University of Amsterdam, Amsterdam, The Netherlands

**Keywords:** Offspring sex ratio, Fisher’s Principle, Heritability, Simulation

## Abstract

Fisher’s principle proposes that the population sex ratio remains approximately equal (around 1:1) through a process of negative frequency-dependent selection. However, large-scale studies show that human offspring sex ratio (OSR) is not heritable in humans, raising doubts about whether Fisher’s principle is a valid explanatory framework for human OSR evolution. Song and Zhang recently proposed a mechanism where OSR propensity is genetically influenced but produces an OSR phenotype with negligible observable heritability due to probabilistic sex determination and small family sizes. Here, we conducted an evaluation of this proposal by simulating the proposed mechanism under a broader range of assumptions, revealing that it only produces undetectable OSR heritability under a specific set of parameters. We further explored whether Fisher’s principle could operate under such low OSR heritability and found, by reanalysing simulation outcomes, that there is only a very narrow parameter space where Fisher’s principle operated and OSR heritability was realistically low. We also make multiple corrections to Song and Zhang’s original analyses which weaken their claim. In sum, our assessment is that evidence for a genetic mechanism that yields undetectable OSR heritability, but allows the operation of Fisher’s Principle, is weak. The proposed mechanism lacks sufficient evidence to overturn the simpler conclusion that OSR is not heritable in humans, and consequently that Fisher’s principle is not a valid explanatory framework for human OSR.

Fisher’s principle, a foundational concept in evolutionary biology, posits that the population sex ratio remains approximately equal through a process of negative frequency-dependent balancing selection. Recent evidence has suggested that human offspring sex ratio (OSR) is not heritable (Boraska et al. 2012; Zietsch et al. 2020), challenging the explanatory power of Fisher’s principle in humans.

OSR is defined as the observed proportion of male offspring produced by an individual, which is the result of a finite number of binary outcomes (individual offspring sexes). Song and Zhang (2024) recently proposed a mechanism where an individual’s latent propensity for OSR (hereafter referred to as OSR propensity) may in fact have a genetic basis, such that Fisher’s principle could operate, but that the heritability of the OSR itself is uniquely low because probabilistic sex determination introduces substantial randomness, especially with small numbers of offspring. This mechanism was demonstrated with a simulation using a transformed distribution of human height as a proxy for OSR propensity, showing that a highly heritable OSR propensity could result in an OSR phenotype with undetectable heritability. They also identify one SNP and two genes associated with OSR using genome-wide association (GWAS) and use hypothetical power analyses to show that even enormous datasets, such as the entire Swedish population, are underpowered to detect such small effects using family designs. Finally, simulation models of Fisher’s principle were used to argue that Fisher’s principle remains a viable explanatory framework for human OSR because, under some parameter conditions, simulation heritability estimates were consistent with empirical heritability estimates.

Here, we provide a critique of this novel and plausible mechanism explaining the lack of OSR heritability – that the underlying OSR propensity is genetically influenced but results in an OSR phenotype with negligible heritability due to probabilistic sex determination and small family sizes. While this would be unusual, as most traits expressed through binary outcomes do exhibit detectable heritability under a liability-threshold model (Falconer and Mackay 1996), it seems plausible that OSR could be expressed through a unique probability-based mechanism, such as the ratio of X to Y sperm production. However, after further evaluating the claim, we argue that the evidence supporting such a genetic mechanism is weak and we remain unconvinced that Fisher’s principle could operate under such conditions. First, we correct a critical error in Song and Zhang’s (2024) simulated power analysis for Zietsch et al. (2020), where they incorrectly applied an adjusted *p*-value threshold of α = 5 × 10⁻⁸ instead of the appropriate α = 0.05. Second, we critique the evidence given in support of the proposed mechanism, questioning the robustness of the SNP finding and demonstrating that the results of the simulations rely heavily on the specific distribution used to simulate OSR propensity. Finally, we challenge the assertion that Fisher’s principle could function with such low OSR heritability, noting that reported simulations indicate only a narrow range of circumstances where this might be possible. Ultimately, we conclude that Song and Zhang’s proposed mechanism is unlikely to result in undetectable OSR heritability while still enabling Fisher’s principle to operate, with limited evidence supporting this explanation over the simpler conclusion: that OSR is not heritable in humans and therefore that Fisher’s principle is not a valid explanatory framework for human OSR. Supporting code and data can be found at https://github.com/ralkuj/SexRatioBehaviorGenetics.

## Power Analysis of Familial OSR Heritability

We begin by addressing an error in Song and Zhang’s (2024) power analysis emulating the Zietsch et al. (2020) analysis. Using Swedish population records, Zietsch et al. analysed 14,015,421 cousin pairs, finding no significant association between an individual’s offspring sex and their sibling’s offspring sex using logistic regression (OR ≈ 1), and estimating zero offspring sex ratio heritability based on tetrachoric correlations (95% CI: [− 0.00076, 0.00196]). The analysis estimated a statistical power of below 0.1% in simulations with a minor allele frequency (MAF) = 10%, allele effect size = 0.10, and α = 0.05, which implies a higher detection rate under the null hypothesis (5%) than under the alternative hypothesis (0.1%) – an impossible scenario. Upon reviewing the simulation code, we identified that a genome-wide significance threshold of α = 5 × 10⁻⁸ was used to determine a positive result. While this α-level adjustment was appropriate for the other GWAS power analyses reported, which must correct for the millions of tests conducted across the genome, it was inappropriate for the familial aggregation analysis in Zietsch et al., where no multiple testing corrections were needed – the correct threshold should have remained at α = 0.05.

**Fig. 1:**
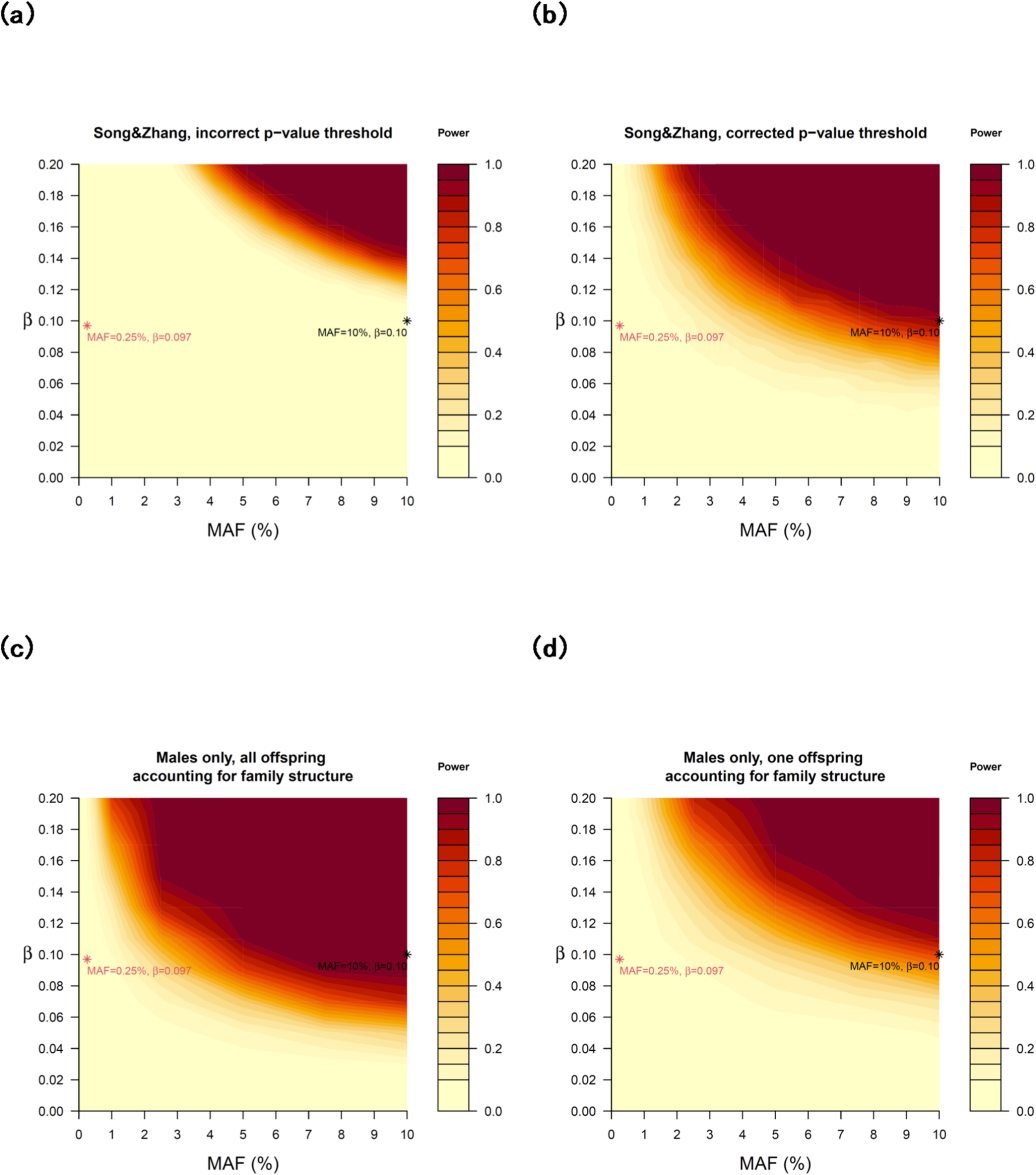
Statistical power simulations based on one genetic locus having influence on sex ratio. **a** Replication of original, incorrect, power calculation by Song and Zhang, using erroneous p-value threshold of 5× 10^− 8^ (14,015,421 cousin pairs). **b** Power calculation with simulation setup the same way as Song and Zhang, but with correct p-value threshold of 0.05 (14,015,421 cousin pairs). **c** Simulation using only male-male sibling parents and all their offspring based on data and methodology in Zietsch et al., p-value threshold 0.05 (2,840,847 cousin pairs). **d** Simulation using only male-male sibling parents and one offspring per father based on data and methodology in Zietsch et al., p-value threshold 0.05 (539,731 cousin pairs). MAF=minor allele frequency; β = effect size of minor allele, i.e., shift in probability of sex of offspring

In Fig. 1 we provide the original Zietsch et al. power analysis reported by Song and Zhang (Fig. 1a) and the corrected power analysis using the threshold of α = 0.05 (Fig. 1b). In the corrected analysis, the example condition of MAF = 10% and allele effect size = 0.10 yielded a power of 90%, rather than 0.1%. Much lower MAFs and/or effect sizes remained practically undetectable, including the SNP with a MAF of 0.25% and effect size of 0.097 identified in the original GWAS. We further updated the simulations by removing the unnecessary randomness in parental sexes, as these are known in the Zietsch et al. data, and by fixing the proportion of males to the observed 51.4%. We also utilized the known familial links and, in line with the original analysis, used cluster robust sandwich estimator for standard errors in a generalized estimating equation model, clustered on sibling clusters, to deal with dependencies between rows of data (i.e., the siblings). We provide one analysis using all offspring from male-male full siblings, which provided realistic power estimates under the assumption of heritable relative proportion of male vs. female gametes produced in males’ spermatogenesis (Fig. 1c), and another using only one offspring per father of male-male sibling pairs, serving as a lower bound (Fig. 1d). These corrected power analyses still reflect the inherent limitations of even the largest datasets in detecting very small effects; however, they reduce the space where OSR heritability could go undetected by existing analyses, narrowing the theoretical conditions under which human OSR could be subject to Fisher’s principle while exhibiting observed heritability indistinguishable from zero.

### Simulated OSR Heritability Under Probabilistic Sex Determination

The proposed low heritability mechanism was originally demonstrated by using a residualized and re-scaled distribution of human height as a substitute for OSR propensity and simulating the resulting OSR phenotype’s heritability (Song and Zhang 2024). Although the simulations showed that a highly heritable underlying trait, expressed through binary outcomes, can result in an observable trait with reduced heritability, these simulations cannot provide evidence that this is the case for human OSR. Song and Zhang performed 20 simulation replicates and applied a Bonferroni correction to their significance threshold. It is unclear whether this correction was applied across the 20 replicates or at a GWAS-wide level, but either approach would be inappropriate. Correcting across the 20 replicates treats them as independent tests, rather than repeated realizations of the same hypothetical analysis, while applying a GWAS-wide correction treats SNP-based heritability as a collection of per-SNP tests rather than a single result. When reassessing their height simulation data at the standard α = 0.05, five of their 20 replicates were significant, compared to the zero reported originally. Further, the choice to use only the distribution of human height, a trait with biological and statistical properties distinct from OSR, lacks justification. The generalisability of the original analysis was greatly constrained by only modelling the one height-based distribution of underlying OSR propensity (i.e. coefficient of variation = 0.05, corresponding standard deviation = 0.025). Notably, the coefficient of variation of height is unusually small among human complex traits (Miller and Penke 2007), so using its distribution as the one stand-in for OSR propensity biases the power analysis in favour of the authors’ conclusions.

We extended the original approach by applying the family structure from Zietsch et al.’s analysis and testing a range of distributions and heritabilities for latent OSR propensity. We assumed probability of a specific sex to be a normally distributed value, with a proportion of variance explained by additive genetics according to a specified heritability value, and the remainder representing unique, non-shared variation. We then drew children according to the family structure observed in Zietsch et al. for male-male parent full sibling pairs only (male sibling pairs being fewer than female sibling pairs) and performed familial aggregation analyses identical to that of Zietsch et al.

Power depended strongly on both the heritability of the latent OSR propensity and the standard deviation of the underlying distribution (Table 1). Across heritabilities, the lowest standard deviation produced low power, reaching 29% when heritability was 100%, while the largest standard deviation value yielded near 100% power even at moderate heritabilities. These findings extend the original approach and demonstrate its sensitivity to parameter values. While the proposed mechanism appears theoretically possible, it is not guaranteed that OSR, being the result of an underlying OSR propensity expressed through probabilistic binary outcomes, would exhibit low heritability; this largely depends on the heritability and variance of the underlying distribution.

**Table 1:** Statistical power to detect an association in the Zietsch et al. study under assumed heritable latent OSR under different simulated scenarios for the OSR

Standard deviation	Heritability 20%	Heritability 40%	Heritability 60%	Heritability 80%	Heritability 100%
− 20%	0.059	0.079	0.113	0.188	0.290
Original distribution	0.071	0.131	0.250	0.388	0.556
+ 20%	0.094	0.225	0.426	0.696	0.843
+ 50%	0.164	0.469	0.819	0.958	0.998
+ 100%	0.357	0.916	1.000	1.000	1.000

Power calculations based on male-male full sibling pairs’ offspring using family structure and analysis approach from Zietsch et al. (2,840,847 cousin pairs). Power calculated from analysis of familial aggregation of sex, i.e. logistic regression with cluster robust sandwich estimator, clustered on extended family identifiers, for standard errors in generalized estimation equations. Results are reported across a range of heritability values of the latent OSR propensity and standard deviation values of the liability distribution (percentages refer to differences in the standard deviation values relative to that used by Song and Zhang)

## Genetic Evidence

Song and Zhang’s (2024) OSR GWAS using the UK Biobank identified a significant SNP on Chromosome 10 (rs144724107; *p* = 3.36 × 10⁻⁸). The low-frequency A allele (0.25%) was associated with a reduced male-to-female ratio, with GA and AA individuals showing 19% and 36% lower OSR, respectively, compared to GG individuals. In genome-wide association studies, true signals typically demonstrate clusters of correlated SNPs due to linkage disequilibrium, while isolated hits are often indicative of noise or statistical artifacts. The identification of a single SNP associated with OSR should not be considered strong evidence, given the Manhattan plot shows no nearby SNPs in linkage disequilibrium with it. Such a result would require strong replication before it could be considered reliable evidence for a genetic basis of OSR.

When evaluating the genetic evidence for OSR heritability, we should also consider how the trait may have been shaped by evolutionary pressures. Fisher’s principle is an example of negative frequency-dependent balancing selection, in which selection changes in direction and magnitude depending on the frequency of the trait in the population. At equilibrium, there is no selection at all (Bull and Charnov 1988). Balancing selection is typically characterised by stable internal equilibria that tend to pull allele frequencies away from the boundaries of loss and fixation, maintaining genetic variance by preserving alleles at intermediate frequencies (Bürger 2000; Turelli and Barton 2004). A SNP as rare as the one identified by Song and Zhang (2024), while possible, would not fit usual expectations for a variant maintained by negative frequency-dependent selection via Fisher’s principle.

## Implications for Fisher’s Principle

The largest existing empirical studies estimate that human OSR has zero heritability (Boraska et al. 2012; Zietsch et al. 2020) – a result fundamentally incompatible with current or past operation of Fisher’s principle (Harper et al. 2024), which poses a significant challenge to its validity as an explanation for human OSR. To address this, it has been proposed that OSR heritability might be so low as to be undetectable in existing analyses, yet still sufficient for Fisher’s principle to function (Song and Zhang 2024). This hypothesis has was tested by simulating 800,000 years of human evolution, adjusting the parameters mutation rate and mutation size for alleles affecting offspring sex propensity. Under some parameter conditions, the resultant simulated OSR heritability fell within the 95% confidence interval of the empirical OSR heritability estimate. Based on these findings, it was concluded by the authors that the observed genetic architecture of human sex ratio is compatible with Fisher’s principle. However, as we discuss further below, it was not adequately determined whether Fisher’s principle operated across all conditions of these models, despite this being central to the conclusions.

**Fig. 2:**
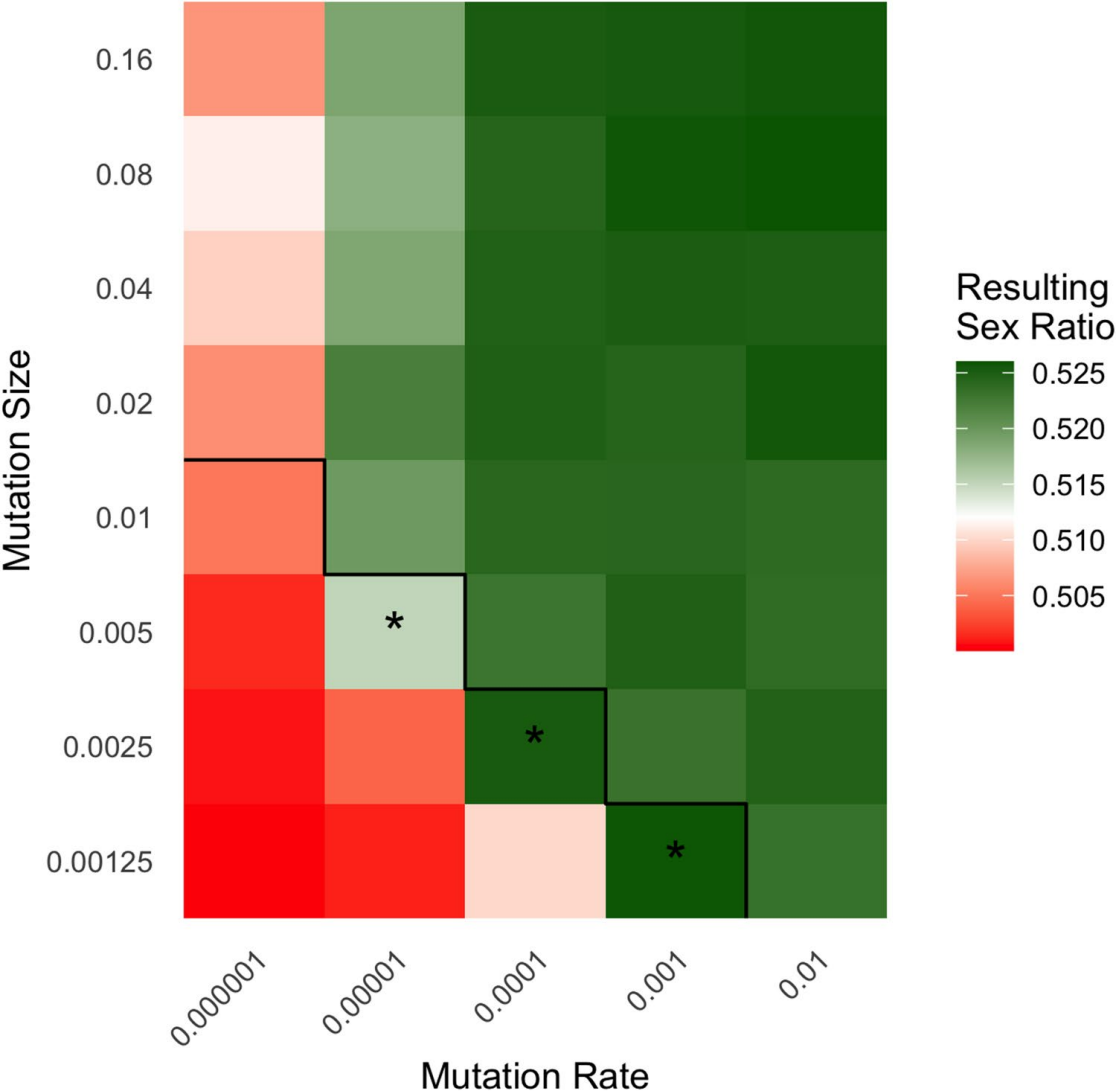
Song and Zhang’s Fisher’s principle simulation results from their “directional” model, with colour indicating the resulting sex ratio for each combination of mutation size and mutation rate parameters. Black line indicates the upper 95%CI of Song and Zhang’s observed heritability, cells underneath this line are parameter conditions where the resulting OSR heritability fell within the 95%CI of empirically observed OSR. White represents the halfway point between the minimum and maximum OSR values. Asterisks indicate conditions where substantial sex ratio evolution occurred and OSR heritability fell within the 95%CI of empirically observes OSR

The lack of observable OSR heritability indicates a non-existent or very weak relationship between genetic variance and phenotypic variance (i.e. between the underlying OSR propensity and the actual OSR). This is not a matter of measurement error, as it has been previously described, but an inherent feature of the genotype-phenotype expression Song and Zhang propose (i.e. probabilistic sex determination and small offspring numbers). OSR propensity is a latent variable which cannot itself be observed, nor is it the quantity for which OSR heritability is estimated in empirical studies, nor is it visible to selection. It is therefore essential to evaluate whether the OSR phenotype, with undetectable heritability reflecting a genuinely weak genetic influence, can still be visible to selection via Fisher’s principle. Here, we examine results from Song and Zhang’s “directional” model where the genetic OSR propensity began at 0.50 and was expected to evolve toward an elevated optimal level via Fisher’s Principle due to unbalanced parental costs of male/female offspring. Figure 2 illustrates the extent to which OSR evolved under each combination of the parameters mutation rate and mutation size, with results coded by colour: white represents conditions where OSR evolved halfway to the purportedly optimal value, red less than halfway, and green more than halfway. Crucially, parameter combinations underneath the black step line indicate where simulated OSR heritability fell within the observed 95% confidence interval of the empirical OSR heritability. Specifically, this criterion was met if the mean of the simulated OSR heritability for a given condition fell within −0.00147 and 0.00038 (the lower and upper confidence intervals from the UK-based GWAS by Song and Zhang).

Of the 40 conditions tested, only three (indicated in Fig. 2 with asterisks) satisfied both criteria of Song and Zhang’s (2024) hypothesis: substantial evolution of OSR via Fisher’s Principle and resulting OSR heritability consistent with empirical estimates. This leaves a very small window of possibility for heritability to be low enough that it is undetectable while also leaving OSR visible to selection (allowing Fisher’s principle to operate). Note that we cannot know the true values of the relevant parameters in humans – we know the human mutation rate per nucleotide, but the mutation rates here (and in Song and Zhang’s (2024) simulations) depend on the mutational target size of OSR^1^, which we do not know. The point, though, is that in a wide parameter space, there is only a small space that is consistent with Song and Zhang’s (2024) basic argument, and there is no reason to believe that the true parameter conditions would lie in this space. Indeed, there are reasons to think they would not.

First, complex traits tend to be associated with many thousands of variants (Zhang et al. 2018; O’Connor et al. 2019), indicating very large mutational target sizes. Small mutational target sizes (and thus low mutation rates in these simulations) are more likely to result in OSR heritability within the 95% confidence intervals of the empirically observed OSR. Second, common variants associated with complex traits generally have very small effect sizes whereas rare variants frequently have large effect sizes – the only known explanation for this pattern is negative selection preventing mutations with large effect from rising to substantial frequency. In contrast, OSR would be under balancing selection (via Fisher’s principle) which would have the opposite effect of keeping variants (with effects large or small) at intermediate frequencies (Bürger 2000; Turelli and Barton 2004). Large effect sizes are not consistent with the range of parameters consistent with Song and Zhang’s (2024) arguments. Third, although we use the upper 95% confidence interval as an upper limit for plausible heritability estimates, the actual estimates for OSR heritability from both Song and Zhang’s (2024) SNP-based estimate and Zietsch et al.’s (2020) family-based estimate are effectively zero, which is unambiguously incompatible with Fisher’s principle. While Song and Zhang’s original simulations are a useful exercise in exploring the theoretical boundaries of Fisher’s Principle under low OSR heritability, their findings ultimately reveal how limited this possibility is.

## Conclusion

Fisher’s principle proposes that the population sex ratio remains approximately equal through a process of negative frequency-dependent selection. However, large-scale studies show that human OSR is not heritable in humans, raising doubts about whether Fisher’s principle is a valid explanatory framework for human OSR evolution. We evaluated the plausibility of a proposed mechanism in which an underlying heritable OSR propensity produces an OSR phenotype with undetectable heritability due to probabilistic sex determination and small family sizes (Song and Zhang 2024). We tested this hypothesis using large-scale simulations, power analyses, and a broader exploration of the parameter ranges used in prior work. We found that the proposed mechanism produces undetectable OSR heritability only under a very specific set of simulation parameters; when OSR is undetectable, Fisher’s principle operates only under rare and unlikely conditions. We conclude that the proposed low heritability mechanism is unlikely to result in undetectable OSR heritability while still enabling Fisher’s principle to operate. The hypothesis lacks sufficient evidence to overturn the simpler conclusion that OSR is not heritable in humans and therefore that Fisher’s principle is not a valid explanatory framework for human OSR.

## Data Availability

Supporting code and data can be found at https://github.com/ralkuj/SexRatioBehaviorGenetics.

## Footnotes

1 That is, the number of loci that, if mutated, would affect OSR propensity.
